# Use of complementary medicine and uptake of COVID-19 vaccination among US adults

**DOI:** 10.3389/fmed.2025.1474914

**Published:** 2025-06-11

**Authors:** Holger Cramer, Mirela Bilc

**Affiliations:** ^1^Institute of General Practice and Interprofessional Care, University Hospital Tübingen, Tübingen, Germany; ^2^Bosch Health Campus, Stuttgart, Germany

**Keywords:** COVID-19, flu, influenza, vaccine, complementary medicine, NHIS

## Abstract

This study investigated the association between complementary medicine (CM) use and the uptake of coronavirus disease 2019 (COVID-19) and flu vaccines in a nationally representative US sample. A secondary analysis of the 2022 National Health Interview Survey data indicated that, after accounting for potential confounders, overall use of CM was not a significant predictor of COVID-19 (*p* = 0.745) or flu vaccination uptake (*p* = 0.123). However, vaccination uptake was lower for both COVID-19 and flu vaccines, respectively, in individuals who visited chiropractors (AOR = 0.78, 95% CI [0.69, 0.89], *p* < 0.001; AOR = 0.71, 95% CI [0.63, 0.81], *p* < 0.001) and naturopaths (AOR = 0.66, 95% CI [0.51, 0.86], *p* = 0.002; AOR = 0.72, 95% CI [0.55, 0.94], *p* = 0.017). Uptake rates for both COVID-19 and flu vaccines were higher among individuals who visited an acupuncturist (COVID-19: AOR = 1.46, 95% CI [1.15, 1.86], *p* = 0.002; flu: AOR = 1.32, 95% CI [1.08, 1.63], *p* = 0.008). The use of mind–body medicine was associated with increased likelihood of COVID-19 vaccination uptake (AOR = 1.24, 95% CI [1.08, 1.42], *p* = 0.002), but not flu vaccination (*p* = 0.264). Visiting a massage therapist was not a significant predictor of either COVID-19 or flu vaccine uptake (*p* = 0.128 and *p* = 0.232, respectively). Overall, the pattern of associations between CM use and COVID-19 vaccination uptake was comparable to that of flu vaccination uptake.

## Introduction

While being one of the most significant public health measures, the success of vaccination largely depends on public acceptance. Vaccine hesitancy has persisted alongside the development of new vaccines, and various controversies, such as those surrounding the smallpox, polio, or MMR vaccines, have fueled public mistrust in vaccine safety and efficacy (1), leading the World Health Organisation (WHO) to recognize vaccine hesitancy as one of the top 10 global health threats (2). Vaccine hesitancy has reached new levels in the COVID-19 era with some specific factors playing an important role: beliefs that vaccines are not safe or effective, increased concerns about the rapid development of COVID-19 vaccines, distrust in medical companies and governments, as well as misinformation and conspiracy theories regarding the origin of the virus or measures such as lockdowns and mandatory vaccinations (3, 4). Social media has played a pivotal role in the so-called COVID-19 infodemic (5).

A recent meta-analysis has shown that conspiracy theories about COVID-19 were negatively associated with attitudes toward vaccination and social distancing, whereas the effects were not significant for mask-wearing and frequent hand-washing. In addition, stronger conspiracy beliefs were associated with a positive attitude toward complementary medicine (CM), while the effects were weaker for actual self-reported behavior (6). Mistrust of vaccine benefit and lower perceived seriousness of COVID-19 were the main determinants of vaccine hesitancy, evaluated as willingness to get vaccinated should a vaccine become available (7). Lower trust in COVID-19 information sources (i.e., medical doctors, scientists, news media, and authorities) and more positive attitudes to CM were also shown to be negatively associated with vaccine intentions (i.e., before a vaccine was available) (8).

Previous studies investigating associations between the use of CM and vaccination uptake have highlighted the complexity of this relationship and the importance of distinguishing between different CM modalities and maybe even between different vaccine types (9, 10). Looking more closely at the flu vaccine as a potential indicator for the COVID-19 vaccination uptake reveals partially divergent findings. One of the first initiatives to analyze this issue using a nationally representative US sample investigated the associations between overall use of CM and flu vaccination rates among adults who were considered a priority for vaccination and those not considered a priority (11). Results showed higher flu vaccination uptake among recent CM users than among those who had never used CM; however, differences between different CM modalities remained unclear. Some of the following studies have shown that individuals consulting chiropractors were less likely to receive the flu vaccine (12), while others reported no significant differences (13, 14). Regarding childhood vaccination uptake, a national survey conducted in Australia found that visits to naturopaths, chiropractors, homeopaths, and traditional Chinese medicine practitioners were weakly to moderately associated with children not having an up-to-date vaccination status (15). Additional data from a nationally representative US sample showed that children who had ever used complementary and manipulative or body-based therapies, such as acupuncture, naturopathy, homeopathy, or chiropractic manipulation, had lower odds of receiving a flu vaccination compared to those who had never used these therapies. In contrast, no significant association was observed with the use of mind–body practices such as yoga, meditation, and progressive relaxation (16). This pattern of partially inconsistent findings may be attributed to methodological differences, including variations in sample composition (e.g., criteria for defining vaccine priority groups), comparison groups (e.g., non-chiropractor users vs. non-CM users), assessment time points (e.g., ever users vs. recent users), and the inclusion of different confounders.

A previous analysis from our group investigated associations between recent use of CM and flu vaccination in the general adult population, as opposed to priority adults only, and found that individuals who visited chiropractors and naturopaths were less likely to get the flu vaccine, while other complementary medicine approaches showed no significant association (17). While this may serve as a better proxy, since the COVID-19 vaccine was recommended for the general population to achieve herd immunity, it remains unclear whether these findings are directly applicable to the uptake of the novel COVID-19 vaccination. Given the high relevance for public health, our objective was to examine the associations between the use of various CM modalities and the uptake of COVID-19 vaccination, and to compare these with associations observed for flu vaccination, using data from the 2022 National Health Interview Survey (NHIS).

## Methods

The NHIS is an annual nationally representative survey of the US civilian non-institutionalized adult population. The NHIS is approved by the Research Ethics Review Board of the National Center for Health Statistics and the US Office of Management and Budget. Secondary analyses of this dataset did not require further IRB review. All NHIS respondents provided oral consent prior to participation. For this study, we analyzed data from *n* = 27,651 adults included in the 2022 NHIS Sample Adult File (see Supplementary S1 file). This publicly available, de-identified dataset was accessed for research purposes on 25 July 2023. The authors had no access to information that could identify participants during or after data collection.

The NHIS uses geographically clustered sampling to ensure cost-effectiveness and national representativeness throughout the year (18). The United States is divided into 1,689 geographical areas, with some states further stratified by population density. Within each stratum, clusters of approximately 2,500 addresses are defined, and a systematic sample of clusters is selected proportionally. Smaller states and the District of Columbia receive more clusters to ensure minimum coverage. Selected households receive a letter and are typically interviewed in person. The sample adults are randomly selected from one of the adults in the selected households. The 2016–2025 NHIS sampling design aims to complete approximately 27,000 adult and 9,000 child interviews annually. In the NHIS 2022, a total of n = 27,651 adults were interviewed from 31,579 eligible sample adults. Because not all selected individuals participate, sampling weights—adjusted for design, non-response, and calibration—are applied to produce nationally representative estimates. The weight for a given respondent stands for the number of persons in the NHIS target population that the respondent represents.

Our main variables of interest were the uptake of COVID-19 and flu vaccination (yes/no), as well as use of CM in the past 12 months (yes/no). Since no direct question was asked about the COVID-19 vaccination within the past 12 months, we computed this indirectly for those who reported ever receiving the vaccine, based on the interview month and the reported month of their most recent COVID-19 vaccination. A direct question was available for the flu vaccination. The use of complementary medicine in the past 12 months included practitioner-based modalities such as visits to a chiropractor, acupuncturist, massage therapist, naturopath, art and/or music therapist, and mind–body medicine, including meditation, guided imagery, progressive relaxation, and yoga. We also computed an overall measure referred to as any CM use—coded as ‘yes’ if participants reported using at least one or more CM modalities and coded as ‘no’ if participants did not use any CM in the past 12 months. No further distinctions were made based on the number of CM modalities used (e.g., one vs. two vs. three, etc.). We additionally extracted data on the following socio-demographic and clinical characteristics associated with potential flu-and COVID-19-related complications: age (less than 65; 65 or older), sex (male; female), ethnicity (Non-Hispanic White; Hispanic; African-American; Asian; Other) education (college or more; less than college), marital status (unknown; married or living with partner; neither married, nor living with partner), household region (Northeast; Midwest; South; West), urban/rural (large metropolitan area; medium and small metropolitan area; non-metropolitan); ratio of income to poverty (not in poverty; in poverty), currently providing or volunteering in health care (yes; no; unknown), health insurance status (private; public – i.e., Medicaid, Medicare or other public; not covered), prior diagnosis of hypertension, coronary heart disease, angina, heart attack, stroke, asthma, cancer, diabetes, COPD, emphysema or chronic bronchitis, dementia, anxiety, depression (yes; no), general health status (excellent or very good; good or fair; poor), weight (healthy weight; underweight; overweight; obese; unknown), current or recent pregnancy (yes, no, unknown or non-applicable), weakened immune system (yes; no), disability (yes; no), current smoking status (yes; no), and previous positive COVID-19 test (yes; no, unknown).

We used NHIS weights to report nationally representative estimates of COVID-19 and flu vaccination uptake and CM use. We used regression analyses to predict the uptake of COVID-19 and flu vaccination in the past 12 months based on CM use while accounting for demographic and clinical confounders. Independent socio-demographic predictors of CM use were identified using multiple logistic regression analysis for the overall use of CM. Given the complexity of the NHIS sampling design, the use of standard statistical procedures would overinflate Type 1 error and therefore result in inaccurate estimates. To account for the stratified cluster sampling and accurately estimate the sampling error, nesting variables were used as indicated in the survey description (18). Statistical analyses were performed using the specialized R “survey” package (19).

## Results

### Prevalence estimates and predictors of CM use

Using the NHIS 2022 census-based weights, data collected from *n* = 27,651 adults were representative of 255,371,962 US adults. National representative estimates indicate a prevalence of 63.6% for COVID-19 vaccination and 47.2% for flu vaccination uptake in the past 12 months. Regarding the use of complementary medicine, overall 38.2% of US adults had seen one or more CM practitioners and/or had used mind–body medicine (i.e., any CM use) in the past 12 months. Specifically, 11.3% of US adults visited a chiropractor, 11.1% a massage therapist, 2.3% an acupuncturist, 1.4% a naturopath, 1.0% an art and/or music therapist, and 27.1% used mind–body medicine.

CM use was more likely among respondents under the age of 65 (AOR = 1.58, 95% CI [1.47, 1.70], *p* < 0.001), females (AOR = 1.77, 95% CI [1.65, 1.89], *p* < 0.001), and individuals residing in the Midwest (AOR = 1.20, 95% CI [1.06, 1.34], *p* < 0.003) and Western (AOR = 1.33, 95% CI [1.18, 1.49], *p* < 0.001) regions of the United States. In contrast, lower CM utilization was observed among Hispanic (AOR = 0.64, 95% CI [0.58, 0.70], *p* < 0.001), African American (AOR = 0.72, 95% CI [0.64, 0.80], *p* < 0.001), and Asian (AOR = 0.78, 95% CI [0.68, 0.90], *p* < 0.001) populations; individuals with less than a college education (AOR = 0.45, 95% CI [0.42, 0.49], *p* < 0.001); residents of medium and small sized metropolitan areas (AOR = 0.87, 95% CI [0.80, 0.94], *p* < 0.001) and non-metropolitan areas (AOR = 0.73, 95% CI [0.66, 0.81], *p* < 0.001); those living in poverty (AOR = 0.85, 95% CI [0.76, 0.95], *p* < 0.006); and individuals covered by public insurance (AOR = 0.87, 95% CI [0.81, 0.94], *p* < 0.001) (see Supplementary Table S1 for full regression analysis).

### Differences in CM use depending on vaccination status

**Figure 1:**
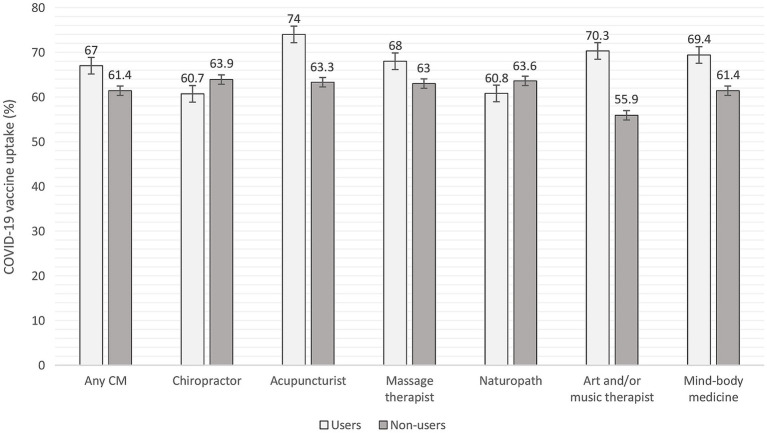
COVID-19 vaccine uptake among users and non-users of complementary medicine assessed in the past 12 months. CM = complementary medicine. Error bars represent standard errors.

**Figure 2:**
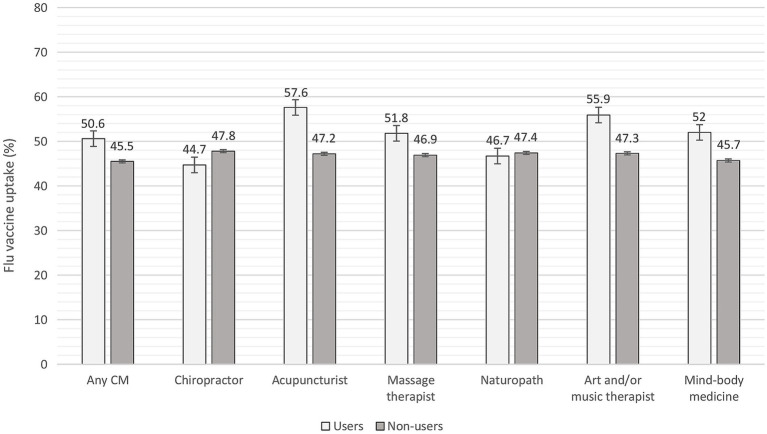
Flu vaccine uptake among users and non-users of complementary medicine assessed in the past 12 months. CM = complementary medicine. Error bars represent standard errors.

Among CM users (i.e., any CM), 67.0% had received the COVID-19 vaccine compared to 61.4% of CM non-users. Uptake of the flu vaccine was 50.6% among CM users compared to 45.5% among CM non-users. Further estimates of vaccination uptake among users vs. non-users of various CM modalities are shown in Figures 1, 2.

The results of the regression analyses (Table 1) have shown that after accounting for potential confounders, the overall use of any CM did not significantly predict uptake of the COVID-19 (*p* = 0.745) or the flu vaccination (*p* = 0.123). Individuals who visited a chiropractor were less likely to receive the COVID-19 (*p* < 0.001) and the flu vaccine (*p* < 0.001). Individuals who visited an acupuncturist were more likely to receive the COVID-19 (*p* = 0.002) or the flu vaccine (*p* = 0.008). Individuals who visited a massage therapist were as likely as non-users to receive the COVID-19 (*p* = 0.128) and the flu vaccine (*p* = 0.232). Individuals who visited a naturopath were less likely to receive the COVID-19 (*p* = 0.002) and the flu vaccine (*p* = 0.017). Individuals who visited an art and/or music therapist were as likely as non-users to receive the COVID-19 vaccine (*p* = 0.132) and the flu vaccine (*p* = 0.057). Users of mind–body medicine were more likely to receive the COVID-19 vaccine (*p* = 0.002), but equally likely as non-users to receive the flu vaccine (*p* = 0.264).

**Table 1:** Regression analysis of complementary medicine use in the prediction of COVID-19 and flu vaccine uptake.

	COVID-19 vaccination status^a^		Flu vaccination status^a^	
CM use^b^	AOR (95% CI)	*p-value*	AOR (95% CI)	*p-value*
Any complementary medicine	1.02 (0.88–1.19)	*p* = 0.745	1.11 (0.97–1.27)	*p* = 0.123
Chiropractor	0.78 (0.69–0.89)	*p* = < 0.001	0.71 (0.63–0.81)	*p* < 0.001
Acupuncturist	1.46 (1.15–1.86)	*p* = 0.002	1.32 (1.08–1.63)	*p* = 0.008
Massage therapist	1.10 (0.97–1.25)	*p* = 0.128	1.07 (0.96–1.20)	*p* = 0.232
Naturopath	0.66 (0.51–0.86)	*p* = 0.002	0.72 (0.55–0.94)	*p* = 0.017
Art and/or music therapist	1.31 (0.92–1.86)	*P* = 0.132	1.39 (0.99–1.95)	*p* = 0.057
Mind–body medicine	1.24 (1.08–1.42)	*p* = 0.002	1.07 (0.95–1.22)	*p* = 0.264

AOR, adjusted odds ratio; CI, confidence interval; AOR after controlling for confounders (see Supplementary Table S1 for full regression analysis).

a Reference is no vaccination.

b Reference is no use.

From the potential confounders that we included the following demographic and clinical variables significantly predicted the uptake of COVID-19 and flu vaccine: age, sex, ethnicity, education, household region, urban/rural, currently providing or volunteering in health care, health insurance, prior diagnostic of hypertension, cancer, depression, current or recent pregnancy, weakened immune system, current smoking status, and previous positive COVID-19 test. Uptake of flu vaccine was additionally predicted by the ratio of income to poverty, prior diagnosis of coronary heart disease, diabetes, and weight (see Supplementary Table S2 for full regression analyses).

## Discussion

This analysis investigated the relationship between CM use and the uptake of COVID-19 and flu vaccination in a nationally representative US sample. The COVID-19 vaccine demonstrated a higher uptake rate (63.6%) compared to the flu vaccine (47.2%.) This difference may be due to COVID-19’s unique combination of urgency (i.e., high perceived risk), policy pressure (e.g., vaccination mandates, incentives, and high accessibility), and public attention (e.g., novelty effect, media coverage, and peer-pressure), all of which may have led to higher uptake relative to the flu vaccine.

While no data from previous studies are available for the novel COVID-19 vaccine, the 47.2% uptake of flu vaccination indicates an increase of 4.5% points compared to 2017 (13), with higher uptake among individuals aged 65 or older (70.5) compared to those under 65 years of age (40.5%). This corresponds to a previously reported general increase in willingness to vaccinate against flu over time (16), as well as CDC recommendations for vaccinations due to the increased risk of developing flu complications for adults over 65 years (20). Our data also show an increase in CM use from 32.4% in 2017 to 38.2% in 2022 (13), even though different survey methods make a direct comparison difficult.

Our results confirm and extend previous findings (12, 13, 17) by showing a pattern of associations between CM use and the uptake of the novel COVID-19 vaccination. While no significant associations emerged using an overall measure of CM use, analyses of different CM modalities revealed significant positive (i.e., visits to an acupuncturist or use of mind–body medicine) and negative (i.e., visits to a naturopath or chiropractor) associations with the uptake of the COVID-19 vaccination. This may reflect broader attitudes toward conventional medicine, where users of acupuncture and mind–body medicine (MBM) are generally more open to conventional health practices such as vaccination that focus on illness prevention. In contrast, those who visit practitioners such as naturopaths and chiropractors may be seeking alternatives to conventional medicine, often favoring natural healing methods over pharmaceutical interventions. The pattern of associations of CM use with COVID-19 vaccination uptake was comparable to that with flu vaccination uptake. This suggests that vaccination hesitancy or advocacy is not specific to the novel mRNA vaccines but more likely represents general health-related beliefs. Furthermore, these findings highlight the need to move beyond overall measures of CM to exploring modality-specific interplays between patients’ beliefs and practitioners’ behavior, which might help better understand vaccination uptake.

While our findings speak against the frequently expressed fear that CM use is a risk factor for vaccine hesitancy per se [e.g., (17, 18)], the intention of use appears to play a role. For instance, in contrast to the US data presented here, a Swiss survey did show a general negative correlation between CM use and uptake of COVID-19 vaccination. However, this correlation was considerably stronger when CM was used to prevent COVID-19, i.e., when it was perceived as an alternative to vaccination despite a lack of evidence (21).

Further considerations involve the potential effects of using CM alongside vaccinations, including how CM might influence the body’s immune response or the overall effectiveness of the vaccine. Mind–body therapies were shown to reduce some types of inflammatory responses and also increase the immune system’s response following vaccination; however, these effects vary depending on the specific biomarkers assessed and the populations studied (22). Several traditional herbal compounds have also been investigated in terms of their potential for developing antiviral agents (23), increasing vaccine efficiency (24, 25), or managing the side effects of vaccination (26).

The results presented in this report are subject to certain limitations. The NHIS survey is an annual cross-sectional survey that includes self-report data of non-institutionalized US residents. In 2022, only a limited number of complementary therapies were assessed. Some important categories, such as the use of dietary supplements or herbal medicine, were not included. In addition, no cross-validation was performed using medical records. There was also no information on the reasons for consulting a CM practitioner, which appears to moderate the relationship between CM use and the uptake of vaccination (19). Due to the cross-sectional design of the NHIS, the results only suggest an association between the use of certain complementary medicine practices and vaccination uptake, but not a causal relationship. For the term “naturopath,” visits to whom were associated with lower vaccination uptake in both this and previous studies (5, 13), there is no uniform definition regarding the complementary therapies employed. Some data include anthroposophic therapies, others focus only on herbal medicine. A similar issue applies to acupuncturists: in the US, some acupuncturists are trained in traditional Chinese medicine, which includes not only acupuncture but also numerous non-pharmacological and pharmacological therapeutic approaches.

Considering the increasing use of complementary medicine, it is of high importance for public health to address both the opportunities and the barriers related to CM use in reaching immunization goals. CM practitioners in support of vaccination are a resource in reaching undecided or vaccine-hesitant patients who might be reluctant to “conventional” information sources. Potential barriers such as personal beliefs, lack of evidence-based knowledge, or lack of communication skills could be addressed by training programs targeting specific groups of CM practitioners. Given that visits to chiropractors were among the most frequently used CM modalities, targeting this group might be particularly promising for policymakers. Providing CM practitioners with evidence-based knowledge, communication training, and tools such as decision aids could contribute to more immunization-related conversations and vaccination-related informed decision-making.

## Data Availability

Publicly available datasets were analyzed in this study. This data can be found at: https://www.cdc.gov/nchs/nhis/2022nhis.htm.

## References

[1] Nuwarda RF Ramzan I Weekes L Kayser V. Vaccine hesitancy: contemporary issues and historical background. Vaccines. (2022) 10:595. doi: 10.3390/vaccines10101595, PMID: 36298459 PMC9612044

[2] World Health Organzation Ten threats to global health in 2019 (2019) Available online at: https://www.who.int/news-room/spotlight/ten-threats-to-global-health-in-2019 (Accessed July 21, 2024).

[3] Aw J Seng JJB Seah SSY Low LL. COVID-19 vaccine hesitancy-a scoping review of literature in high-income countries. Vaccines. (2021) 9:900. doi: 10.3390/vaccines9080900, PMID: 34452026 PMC8402587

[4] Chevallier C Hacquin AS Mercier H. COVID-19 vaccine hesitancy: shortening the last mile. Trends Cogn Sci. (2021) 25:331–3. doi: 10.1016/j.tics.2021.02.002, PMID: 33618982 PMC7871796

[5] The Lancet Infectious Diseases. The COVID-19 infodemic. Lancet Infect Dis. (2020) 20:875. doi: 10.1016/S1473-3099(20)30565-X, PMID: 32687807 PMC7367666

[6] Bierwiaczonek K Gundersen AB Kunst JR. The role of conspiracy beliefs for COVID-19 health responses: a meta-analysis. Curr Opin Psychol. (2022) 46:101346. doi: 10.1016/j.copsyc.2022.101346, PMID: 35486966 PMC8978448

[7] Gerretsen P Kim J Caravaggio F Quilty L Sanches M Wells S. Individual determinants of COVID-19 vaccine hesitancy. PLoS One. (2021) 16:e0258462. doi: 10.1371/journal.pone.0258462, PMID: 34788308 PMC8598046

[8] Soveri A Karlsson LC Antfolk J Lindfelt M Lewandowsky S. Unwillingness to engage in behaviors that protect against COVID-19: the role of conspiracy beliefs, trust, and endorsement of complementary and alternative medicine. BMC Public Health. (2021) 21:1–12. doi: 10.1186/s12889-021-10643-w, PMID: 33832446 PMC8027965

[9] Wardle J Frawley J Adams J Sibbritt D Steel A Lauche R. Associations between complementary medicine utilization and influenza/pneumococcal vaccination: results of a national cross-sectional survey of 9151 Australian women. Prev Med. (2017) 105:184–9. doi: 10.1016/j.ypmed.2017.09.009, PMID: 28911953

[10] Wardle J Frawley J Steel A Sullivan E. Complementary medicine and childhood immunisation: a critical review. Vaccine. (2016) 34:4484–500. doi: 10.1016/j.vaccine.2016.07.026, PMID: 27475472

[11] Stokley S Cullen KA Kennedy A Bardenheier BH. Adult vaccination coverage levels among users of complementary/alternative medicine–results from the 2002 National Health Interview Survey (NHIS). BMC Complement Altern Med. (2008) 8:1–8. doi: 10.1186/1472-6882-8-6, PMID: 18294382 PMC2266896

[12] Jones L Sciamanna C Lehman E. Are those who use specific complementary and alternative medicine therapies less likely to be immunized? Prev Med. (2010) 50:148–54. doi: 10.1016/j.ypmed.2009.12.001, PMID: 20005248

[13] Davis MA Smith M Weeks WB. Influenza vaccination among chiropractic patients and other users of complementary and alternative medicine: are chiropractic patients really different? Prev Med. (2012) 54:5–8. doi: 10.1016/j.ypmed.2011.01.007, PMID: 21296107 PMC3130095

[14] Smith M Davis MA. Immunization status of adult chiropractic patients in analyses of national health interview survey. J Manip Physiol Ther. (2011) 34:602–8. doi: 10.1016/j.jmpt.2011.09.001, PMID: 21943651 PMC3586316

[15] Frawley JE Foley H McIntyre E. The associations between medical, allied and complementary medicine practitioner visits and childhood vaccine uptake. Vaccine. (2018) 36:866–72. doi: 10.1016/j.vaccine.2017.12.036, PMID: 29306509

[16] Bleser WK Elewonibi BR Miranda PY BeLue R. Complementary and alternative medicine and influenza vaccine uptake in US children. Pediatrics. (2016) 138:e20154664. doi: 10.1542/peds.2015-4664, PMID: 27940756 PMC5079075

[17] Kohl-Heckl WK Schroeter M Dobos G Cramer H. Complementary medicine use and flu vaccination–a nationally representative survey of US adults. Vaccine. (2021) 39:5635–40. doi: 10.1016/j.vaccine.2021.08.017 34419302

[18] National Center for Health Statistics. National Health Interview Survey (2022) Available online at: https://ftp.cdc.gov/pub/Health_Statistics/NCHS/Dataset_Documentation/NHIS/2022/srvydesc-508.pdf (Accessed July 25, 2023).

[19] Lumley T. Complex surveys: A guide to analysis using R. New Jersey, USA: John Wiley & Sons (2011).

[20] Center for Disease Control. Flu and people 65 years and older (2024). Available online at: https://www.cdc.gov/flu/highrisk/65over.htm (Accessed July 21, 2024).

[21] Nehme M Braillard O Rodondi PY Guessous I. Use of complementary medicine and its association with SARS-CoV-2 vaccination during the COVID-19 pandemic: a longitudinal cohort study. Swiss Med Wkly. (2023) 153:3505. doi: 10.57187/s.3505, PMID: 38579302

[22] Morgan N Irwin MR Chung M Wang C. The effects of min-body therapies on the immune system: meta-analysis. PLoS One. (2014) 9:e100903. doi: 10.1371/journal.pone.0100903 24988414 PMC4079606

[23] Kesheh MM Shavandi S Haeri Moghaddam N Ramezani M Ramezani F. Effect of herbal compounds on coronavirus; a systematic review and meta-analysis. Virol J. (2022) 19:87. doi: 10.1186/s12985-022-01808-z, PMID: 35597998 PMC9123756

[24] Chopra A Chavan-Gautam P Tillu G Saluja M Borse S Sarmukaddam S. Randomized, double blind, placebo controlled, clinical trial to study Ashwagandha Administration in Participants Vaccinated against COVID-19 on safety, immunogenicity, and protection with COVID-19 vaccine-a study protocol. Front Med. (2022) 9:761655. doi: 10.3389/fmed.2022.761655, PMID: 35252231 PMC8888820

[25] Saggam A Limgaokar K Borse S Chavan-Gautam P Dixit S Tillu G. *Withania somnifera* (L.) Dunal: opportunity for clinical repurposing in COVID-19 management. Front Pharmacol. (2021) 12:623795. doi: 10.3389/fphar.2021.623795, PMID: 34012390 PMC8126694

[26] Yoon HC. Herbal medicine use in Republic of Korea to alleviate side effects of COVID-19 vaccines: a cross-sectional study. J Integr Med. (2023) 21:361–8. doi: 10.1016/j.joim.2023.06.002, PMID: 37349213 PMC10249366

