# Supplementary material for: Use of complementary medicine and uptake of COVID-19 vaccination among US adults
Source: Front Med (Lausanne). 2025 Jun 11;12:1474914. doi: 10.3389/fmed.2025.1474914 (PMC12187769; doi:10.3389/fmed.2025.1474914)
Supplement: Supplementary file 1 [file Table_1.docx]

**Supplementary material**

**S1 File. NHIS variable identification**

Age (AGEP_A, AGE65);

Sex (SEX_A);

Ethnicity (HISPALLP_A);

Education (EDUCP_A);

Marital status (MARITAL_A);

Household region (REGION);

Urban-rural (URBRRLL);

Ratio of income to poverty (RATCAT_A);

Currently providing or volunteering in health care (WORKHEALTH_A, WRKHLTHFC_A);

Health insurance (COVER_A, COVER65_A);

Hypertension (HYPEV_A);

Coronary heart disease (CHDEV_A);

Angina (ANGEV_A);

Heart attack (MIEV_A);

Stroke (STREV_A);

Asthma (ASEV_A);

Cancer (CANEV_A);

Diabetes (DIBEV_A);

COPD, emphysema or chronic bronchitis (COPDEV_A);

Dementia (DEMENEV_A);

Anxiety (ANXEV_A);

Depression (DEPEV_A);

General health status (PHSTAT_A);

Weight (BMICAT_A);

Currently or recently pregnant (PREGNOW_A, PREGFLUYR_A);

Weakened immune system (MEDRXTRT_A, HLTHCOND_A);

Disability (DISAB3_A);

Current smoking status (SMKCIGST_A);

Previous positive COVID-19 test (POSTEST_A);

Chiropractor (CHIRO_A);

Acupuncturist (ACU_A);

Massage therapist (MASS_A);

Naturopath (NATUR_A);

Art and/or music therapist (ARTTHPY_A & MUSICTHPY_A);

Mind-body medicine (MEDITATE_A, GIPR_A, YOGA_A);

Any complementary medicine (CM) (CHIRO_A, ACU_A, MASS_A, NATUR_A, ARTTHPY_A, MUSICTHPY_A, MEDITATE_A, GIPR_A, YOGA_A);

COVID-19 vaccine in the past 12 months (SHTCVD191_A, INTV_MON, CVDVAC1M_A, CVDVAC1Y_A);

Flu vaccine in the past 12 months (SHTFLU12M_A).

**Table S1.**

**Logistic regression analysis predicting overall use of complementary medicine**

|  | **CM use** ^a^ |
| --- | --- |
|  | **AOR (95 % CI)** |
| Age |  |
| 65 or older | Reference |
| Less than 65 | 1.58 (1.47 – 1.70) ^***^ |
| Sex |  |
| Male | Reference |
| Female | 1.77 (1.65 – 1.89) ^***^ |
| Ethnicity |  |
| Non-Hispanic White | Reference |
| Hispanic | 0.64 (0.58 – 0.70) ^***^ |
| African-American | 0.72 (0.64 – 0.80) |
| Asian | 0.78 (0.68 – 0.90) ^***^ |
| Other | 1.11 (0.90 – 1.36) |
| Education |  |
| College or more | Reference |
| Less than college | 0.45 (0.42 – 0.49) ^***^ |
| Marital status |  |
| Unknown | Reference |
| Married or living with partner | 1.02 (0.67 – 1.55) |
| Neither married, nor living with partner | 1.03 (0.68 – 1.57) |
| Household region |  |
| Northeast | Reference |
| Midwest | 1.20 (1.06 – 1.34) ^**^ |
| South | 0.90 (0.81 – 1.00) |
| West | 1.33 (1.18 – 1.49) ^***^ |
| Urban/Rural |  |
| Large metropolitan | Reference |
| Medium and small metropolitan | 0.87 (0.80 – 0.94) ^***^ |
| Non-metropolitan | 0.73 (0.66 – 0.81) ^***^ |
| Ratio of income to poverty |  |
| Not in poverty | Reference |
| In poverty | 0.85 (0.76 – 0.95) ^**^ |
| Health insurance |  |
| Private | Reference |
| Public (i.e. Medicaid, Medicare or other public) | 0.87 (0.81 – 0.94) ^***^ |
| Not covered | 0.90 (0.79 – 1.02) |

Note: ^a^ Reference is 0 = no use; CM = complementary medicine, AOR= odds ratio, CI= confidence interval; ^***^ p ≤ 0.001; ^**^ p ≤ 0.01.

**Table S2.**

**Logistic regression analysis predicting COVID-19 and flu vaccine uptake**

|  | **Weighted estimates (%)^a^** | **COVID-19 vaccine^b^** | **Flu vaccine^b^** |
| --- | --- | --- | --- |
|  |  | **AOR (95 % CI)** | **AOR (95 % CI)** |
| Age | | | |
| 65 or older | 22.2% | Reference | Reference |
| Less than 65 | 77.8% | 0.51 (0.46 – 0.56) ^***^ | 0.40 (0.37 – 0.44) ^***^ |
| Sex | | | |
| Male | 48.7% | Reference | Reference |
| Female | 51.3% | 1.29 (1.20 – 1.40) ^***^ | 1.51 (1.39 – 1.64) ^***^ |
| Ethnicity | | | |
| Non-Hispanic White | 62.1% | Reference | Reference |
| Hispanic | 17.2% | 1.30 (1.15 – 1.46) ^***^ | 0.94 (0.85 – 1.05) |
| African-American | 11.9% | 1.00 (0.89 – 1.13) | 0.67 (0.60 – 0.75) ^***^ |
| Asian | 6.0% | 1.99 (1.69 – 2.35) ^***^ | 1.36 (1.18 – 1.56) ^***^ |
| Other | 2.8% | 1.08 (0.89 – 1.32) | 0.99 (0.80 – 1.24) |
| Education | | | |
| College or more | 61.6% | Reference | Reference |
| Less than college | 37.7% | 0.70 (0.65 – 0.76) ^***^ | 0.73 (0.68 – 0.79) ^***^ |
| Marital status | | | |
| Unknown | 4.5% | Reference | Reference |
| Married or living with partner | 57.7% | 0.93 (0.58 – 1.48) | 1.03 (0.66 -1.61) |
| Neither married, nor living with partner | 37.7% | 0.82 (0.51 – 1.30) | 0.77 (0.49 – 1.20) |
| Household region | | | |
| Northeast | 17.6% | Reference | Reference |
| Midwest | 20.7% | 0.82 (0.73 – 0.93) ^**^ | 0.88 (0.80 – 0.98) ^*^ |
| South | 38.1% | 0.66 (0.58 – 0.74) ^***^ | 0.70 (0.63 – 0.78) ^***^ |
| West | 23.6% | 0.94 (0.83 – 1.07) | 0.79 (0.70 – 0.89) ^***^ |
| Urban/Rural | | | |
| Large metropolitan | 56.3% | Reference | Reference |
| Medium and small metropolitan | 29.8% | 0.78 (0.71 – 0.85) ^***^ | 0.92 (0.84 – 1.01) ^*^ |
| Non-metropolitan | 13.8% | 0.61 (0.54 – 0.70) ^***^ | 0.72 (0.64 – 0.80) ^***^ |
| Ratio of income to poverty | | | |
| Not in poverty | 90.2% | Reference | Reference |
| In poverty | 9.8% | 0.89 (0.80 – 1.00) | 0.87 (0.77 – 0.98) ^*^ |
| Currently providing or volunteering in health care | | | |
| No | 87.8% | Reference | Reference |
| Yes | 10.9% | 1.18 (1.05 – 1.31) ^**^ | 2.35 (2.12 – 2.60) ^***^ |
| Unknown | 1.4% | 2.80 (0.31 – 25.48) | 5.95 (1.23 – 28.75) |
| Health insurance | | | |
| Private | 60.5% | Reference | Reference |
| Public (i.e. Medicaid, Medicare or other public) | 29.7% | 0.76 (0.70 – 0.83) ^***^ | 0.92 (0.85 – 1.00) |
| Not covered | 9.8% | 0.53 (0.46 – 0.59) ^***^ | 0.38 (0.33 – 0.44) ^***^ |
| (PD) Hypertension | | | |
| No | 68.0% | Reference | Reference |
| Yes | 32.0% | 1.13 (1.04 – 1.22) ^**^ | 1.37 (1.28 – 1.48) ^***^ |
| (PD) Coronary heart disease | | | |
| No | 95.1% | Reference | Reference |
| Yes | 4.9% | 1.12 (0.93 – 1.35) | 1.29 (1.08 – 1.54) ^**^ |
| (PD) Angina | | | |
| No | 98.4% | Reference | Reference |
| Yes | 1.6% | 1.02 (0.78 – 1.33) | 1.15 (0.86 – 1.53) |
| (PD) Heart attack | | | |
| No | 97.0% | Reference | Reference |
| Yes | 3.0% | 1.03 (0.84 – 1.25) | 1.18 (0.95 – 1.46) |
| (PD) Stroke | | | |
| No | 97.2% | Reference | Reference |
| Yes | 2.8% | 1.05 (0.85 – 1.29) | 1.13 (0.93 – 1.37) |
| (PD) Asthma | | | |
| No | 85.5% | Reference | Reference |
| Yes | 14.5% | 0.99 (0.90 – 1.09) | 1.09 (0.99 – 1.19) |
| (PD) Cancer | | | |
| No | 90.4% | Reference | Reference |
| Yes | 9.6% | 1.19 (1.07 – 1.32) ^***^ | 1.38 (1.24 – 1.52)  ^***^ |
| (PD) Diabetes | | | |
| No | 90.4% | Reference | Reference |
| Yes | 9.6% | 1.11 (0.99 – 1.25) | 1.52 (1.36 – 1.71) ^***^ |
| (PD) COPD, emphysema or chronic bronchitis | | | |
| No | 95.4% | Reference | Reference |
| Yes | 4.6% | 1.01 (0.87 – 1.19) | 1.14 (0.97 – 1.33) |
| (PD) Dementia | | | |
| No | 98.9% | Reference | Reference |
| Yes | 1.1% | 0.92 (0.65 – 1.31) | 0.76 (0.54 – 1.06) |
| (PD) Anxiety | | | |
| No | 82.2% | Reference | Reference |
| Yes | 17.8% | 1.08 (0.97 – 1.21) | 1.10 (0.99 – 1.22) |
| (PD) Depression | | | |
| No | 81.8% | Reference | Reference |
| Yes | 18.2% | 1.13 (1.02 – 1.26) ^*^ | 1.22 (1.09 – 1.36) ^***^ |
| General health status | | | |
| Excellent or very good | 56.0% | Reference | Reference |
| Good or fair | 40.6% | 0.98 (0.91 – 1.06) | 0.96 (0.89 – 1.04) |
| Poor | 3.3% | 1.10 (0.89 – 1.35) | 0.94 (0.76 – 1.17) |
| Weight | | | |
| Healthy weight | 30.6% | Reference | Reference |
| Underweight | 1.6% | 0.83 (0.64 – 1.08) | 0.73 (0.54 – 0.97) ^*^ |
| Overweight | 33.1% | 0.95 (0.87 – 1.04) | 1.03 (0.95 – 1.12) |
| Obese | 32.4% | 0.96 (0.88 – 1.05) | 1.00 (0.92 – 1.09) |
| Unknown | 2.2% | 1.09 (0.85 – 1.39) | 0.79 (0.63 – 1.00) |
| Current or recent pregnancy | | | |
| No | 24.9% | Reference | Reference |
| Yes | 1.9% | 0.52 (0.41 – 0.66) ^***^ | 1.14 (0.90 – 1.44) |
| Unknown or N/A | 73.2% | 1.35 (1.22 – 1.48) ^***^ | 1.38 (1.25 – 1.53) ^***^ |
| Weakened immune system | | | |
| No | 92.6% | Reference | Reference |
| Yes | 7.4% | 1.14 (1.01 – 1.30) ^*^ | 1.31 (1.16 – 1.47) ^***^ |
| Disability | | | |
| No | 90.7% | Reference | Reference |
| Yes | 9.3% | 0.97 (0.85 – 1.11) | 1.06 (0.93 – 1.21) |
| Current smoking status | | | |
| No | 88.7% | Reference | Reference |
| Yes | 11.3% | 0.65 (0.59 – 0.72) ^***^ | 0.56 (0.51 – 0.62) ^***^ |
| Previous positive COVID-19 test | | | |
| No | 61.4% | Reference | Reference |
| Yes | 38.0% | 0.70 (0.65 – 0.75) ^***^ | 0.82 (0.77 – 0.87) ^***^ |
| Unknown | 0.6% | 0.72 (0.21 – 2.51) | 0.58 (0.27 – 1.21) |
| Any complementary medicine | | | |
| No | 61.8% | Reference | Reference |
| Yes | 38.2% | 1.02 (0.88 – 1.19) | 1.11 (0.97 – 1.27) |
| Chiropractor | | | |
| No | 88.7% | Reference | Reference |
| Yes | 11.3% | 0.78 (0.69 – 0.89) ^***^ | 0.71 (0.63 – 0.81) ^***^ |
| Acupuncturist | | | |
| No | 97.7% | Reference | Reference |
| Yes | 2.3% | 1.46 (1.15 – 1.86) ^**^ | 1.32 (1.08 – 1.63) ^*^ |
| Massage therapist | | | |
| No | 88.9% | Reference | Reference |
| Yes | 11.1% | 1.10 (0.97 – 1.25) | 1.07 (0.96 – 1.20) |
| Naturopath | | | |
| No | 98.6% | Reference | Reference |
| Yes | 1.4% | 0.66 (0.51 – 0.86) ^**^ | 0.72 (0.55 – 0.94) ^*^ |
| Art and/or music therapist | | | |
| No | 99.0% | Reference | Reference |
| Yes | 1.0% | 1.31 (0.92 – 1.86) | 1.39 (0.99 – 1.95)^*^ |
| Mind-body medicine | | | |
| No | 72.9% | Reference | Reference |
| Yes | 27.1% | 1.24 (1.08 – 1.42) ^**^ | 1.07 (0.95 – 1.22) |

Note: ^a^ Percentages are based on NHIS weights; ^b^ Reference is 0 = no vaccination;

AOR= odds ratio, CI= confidence interval, PD = prior diagnostic; N/A = not applicable

^***^ p ≤ 0.001; ^**^ p ≤ 0.01; ^*^p ≤ 0.05
